# An Evaluation of the Impact of the Amount of Potassium Hydroxide on the Porous Structure Development of Activated Carbons

**DOI:** 10.3390/ma14082045

**Published:** 2021-04-19

**Authors:** Mirosław Kwiatkowski, Elżbieta Broniek, Vanessa Fierro, Alain Celzard

**Affiliations:** 1 Faculty of Energy and Fuels, AGH University of Science and Technology, al. A. Mickiewicza 30, 30-059 Krakow, Poland; 2 Faculty of Chemistry, Wrocław University of Technology, Gdańska 7/9, 50-344 Wrocław, Poland; elzbieta.broniek@pwr.edu.pl; 3 Unité Mixte de Recherche (UMR), Centre National de la Recherche Scientifique (CNRS), Institute Jean Lamour, Université de Lorraine n°7198, ENSTIB, 27 Rue Philippe Séguin, BP 21042, CEDEX 09, 88051 Epinal, France; vanessa.fierro@univ-lorraine.fr (V.F.); alain.celzard@univ-lorraine.fr (A.C.)

**Keywords:** activated carbons, adsorption, porous structure, biomass-based materials

## Abstract

This paper presents the results of an evaluation of the impact of the amount of potassium hydroxide on the obtained porous structure of the activated carbons derived from the shells of pistachios, hazelnuts, and pecans by carbonization and subsequent chemical activation with potassium hydroxide by different adsorption methods: Brunauer–Emmett–Teller, Dubinin–Raduskevich, the new numerical clustering-based adsorption analysis, Quenched Solid Density Functional Theory, and 2D-Non-linear Density Functional Theory for Heterogeneous Surfaces, applied to nitrogen adsorption isotherms at −196 °C. Based on the conducted research, a significant potential for the production of activated carbons from waste materials, such as nut shells, has been demonstrated. All the activated carbons obtained in the present study at the activator/char mass ratio *R* = 4 exhibited the most developed porous structure, and thus very good adsorption properties. However, activated carbons obtained from pecan shells deserve special attention, as they were characterized by the most homogeneous surface among all the samples analyzed, i.e., by a very desirable feature in most adsorption processes. The paper demonstrates the necessity of using different methods to analyze the porous structure of activated carbons in order to obtain a complete picture of the studied texture. This is because only a full spectrum of information allows for correctly selecting the appropriate technology and conditions for the production of activated carbons dedicated to specific industrial applications. As shown in this work, relying only on the simplest methods of adsorption isotherm analysis can lead to erroneous conclusions due to lack of complete information on the analyzed porous structure. This work thus also explains how and why the usual characterizations of the porous structure of activated carbons derived from lignocellulosic biomass should not be taken at face value. On the contrary, it is advisable to cross reference several models to get a precise idea of the adsorbent properties of these materials, and therefore to propose the most suitable production technology, as well as the conditions of the preparation process.

## 1. Introduction

Activated carbons are materials whose adsorption properties make them indispensable for a large number of applications related to the environment. Indeed, thanks to their highly developed specific surface areas and large micro/mesoporous volumes, they can trap significant quantities of pollutants. Common applications are therefore air purification [1] and water treatment [2,3,4,5,6], but they can also be used in catalysis [7,8,9,10,11], as well as in energy-related applications, e.g., gas storage [12,13,14,15,16,17], solar refrigeration by adsorption [18,19], storage, and the conversion of electrochemical energy [20,21,22,23]. However, not all activated carbons are necessarily efficient in a given application, and it is necessary that their porous texture be optimized for the intended purpose. This raises the very necessary question of their characterization and preparation conditions [24]. Indeed, the final porous texture of activated carbons is dependent not only on the starting precursor, which can be any organic matter—especially biomass [25,26,27,28,29], but also, and above all, on its conditions of transformation into activated carbon [30,31,32,33,34,35]. This process can be carried out in different ways, but always leads to an enrichment of the starting organic matter into carbon (the carbonization stage), and to the opening and development of porosity in the final material (the activation stage). For a long time now, it has been known how to produce activated carbons and how to optimize them for one application or another. However, the new adsorption technologies and their need to compete with other technologies require increasingly efficient adsorbents, and simple physical adsorption methods do not make it possible to obtain them. Therefore, new methods of obtaining them are being sought, and there is great scope for developing chemical activation methods, including the search for new raw materials that could be used in the production of highly effective activated carbons.

Knowledge of activated carbons’ porous textures, which should be directly related to the expected adsorption performance, necessarily involves physicochemical characterizations with which models are associated. Routine characterization includes nitrogen adsorption at the temperature of −196 °C, and the treatment of isotherms is then usually entrusted to models whose limitations are generally—but not always—known by the researchers using them. However, for lack of better models, they continue to be widely used throughout the world. In this work, we aimed to insist on the need for not being satisfied with one or two models, in particular the most usual ones, to characterize activated carbons. Although such common models continue to be extremely useful, particularly for comparative purposes with other data from the literature, we show that the comparison of different and more advanced models allows us to go much further in understanding the porous structure. Thus, we have produced activated carbons derived from food waste, in this case, the shells of various edible nuts, by chemical activation and applied these different models to them. We then show that the usual models do not allow us to clearly distinguish certain activated carbons, but that other models reveal some clear differences. These results should therefore allow for better control of the preparation of carbonaceous adsorbents for given applications, thanks to the very fine characterization made possible by the comparison of different adsorption models.

## 2. Materials and Methods

### 2.1. Preparation of Activated Carbon Samples

Activated carbons (AC) were produced from pistachio nut shells (marked as POAC), hazelnut shells (HTAC), and pecan nut shells (PNAC). For that purpose, a grain fraction of 1–3.15 mm was first separated and then subjected to carbonization at 500 °C under a nitrogen flow of 30 dm^3^/h. The chars thus obtained from each of the raw materials were then mixed with solid potassium hydroxide powder (Sigma-Aldrich Chemie GmbH, Munich, Germany) and crushed in a mortar to homogenize the mixtures. For the purposes of the study, samples of activated carbons were obtained according to different ratios of the mass of the activator to that of the char, i.e., *R* = 1, 2, 3, and 4. In preliminary studies, higher amounts of KOH were also considered; however, rapid degradation of the porous structure occurred for mass ratios above *R* = 4. After mixing with potassium hydroxide, the samples were heated in a vertical tubular furnace under nitrogen flow (30 dm^3^/h) at a rate of 5 °C/min until the final activation temperature of 800 °C. The materials were subsequently held at the final activation temperature for 1 h and then cooled to room temperature under nitrogen flow. The obtained activated carbons were next treated with 0.5 mol/dm^3^ HCl (Sigma-Aldrich Chemie GmbH, Munich, Germany) solution and washed with hot distilled water to wash out the chloride ions. The final stage of preparation of the samples consisted of drying the activated carbons at a temperature of 100 °C to constant weight.

### 2.2. Adsorption Models Considered in This Study

It is well known that activated carbons are characterized by a very complex structural and chemical structure, and therefore their description is problematic despite the development of many analytical methods. These methods take into account different structure models, as well as other aspects relating to the physicochemistry of these materials.

The Brunauer–Emmett–Teller (BET) method [36] is widely adopted for the determination of the specific area of porous materials based on the isothermal adsorption of nitrogen vapor at −196 °C, and its great popularity is mainly due to the simple form of the BET equation. Unfortunately, the assumptions of this method differ significantly from reality, and in particular, the method does not take into account the heterogeneity of surfaces as well as the not taking into account the interactions between the adsorbed molecules. However, and despite many limitations, this method ensures the comparability of results for different materials.

Another very popular method used in the analysis of the porous structure of activated carbons is the Dubinin–Raduskevich (DR) method [37], which is used to determine the volume of micropores. However, this method also has many limitations and, in particular, does not take into account the heterogeneity of the surface of the material under analysis. Therefore, given the limitations of the BET and DR methods, research has proposed methods that are more sophisticated for pore structure analysis. Here, the new numerical clustering based adsorption analysis (LBET) method, together with the implemented unique numerical procedure for the fast multivariant identification of adsorption systems [38,39,40,41], as well as Quenched Solid Density Functional Theory (QSDFT) [42,43] and 2D-Non-linear Density Functional Theory for Heterogeneous Surfaces (2D-NLDFT-HS) [44,45,46] methods were chosen to determine pore size distributions, taking into account, among other things, the heterogeneity of the surface.

Thus, the LBET method described in detail in the authors’ previous works is based on unique mathematical models that take into account, in addition to surface heterogeneity, the possibility of molecule clusters branching and the geometric and energy limitations of adsorbate cluster formation [38,39,40,41].

The LBET models have five parameters: the volume of the first adsorbed layer, *V_hA_* [cm^3^/g]; the dimensionless energy parameter for the first layer, *Q_A_*/*RT*; the dimensionless energy parameter for the higher layers, *B_C_*; the geometrical parameter of the porous structure determining the height of the adsorbate molecule clusters, *α*; and the geometrical parameter of the porous structure determining the width of the adsorbate molecule clusters, *β*, which can be adjusted by fitting the LBET equation to the adsorption isotherm [38,39,40,41]. In addition, the LBET method implements a fast multivariate procedure of adsorption system identification, which allows for the determination of the surface heterogeneity parameter *h* and the shape of the adsorption energy distribution on the first layer. The procedure mentioned above consists of fitting a set of theoretical isotherms corresponding to thirty LBET class models to the experimental adsorption isotherm, covering all possible cases of adsorption energy distributions on the surface of carbon adsorbents, as well as the degree of surface heterogeneity [38,39,40,41]. This approach avoids inadvisable overparameterization of the mathematical models of the adsorption process; simplifies the numerical procedure itself; and, consequently, increases the reliability of the determined parameters of the porous structure.

It should be emphasized that each of the information types obtained by the LBET method, i.e., the values of the model parameters *V_hA_*, *Q_A_/RT*, *B_C_*, *α*, and *β*; the value of the surface heterogeneity parameter *h*; as well as the distribution of adsorption energy on the adsorbent surface, provide valuable information for technologists involved in the production of activated carbons, as well as for scientists studying, among other things, the mechanisms occurring during the preparation of activated carbons [38,39,40,41].

To determine pore size distributions, two methods have been selected that differ in the approach to take into account the heterogeneity of the activated carbon surface. The QSDFT method [42,43] takes into account the heterogeneity of the surface in one dimension, while the 2D-NLDFT-HS method considers it in two dimensions [44,45,46].

These methods are an important development of the basic NLDFT method [47], which adopted a geometrically and energetically ideal homogeneous surface and split-like structure of the pores, ignoring the heterogeneity of the surface and deviations from the ideal split structure of the pores of real microporous materials. Consequently, the theoretical isotherms of NLDFT adsorption have shown numerous steps related to the formation of successive layers of adsorbate. The problem occurred particularly in microporous carbon materials, manifested by artificial breaks in the calculated pore size distributions at about 1 and 2 nm [48].

### 2.3. Textural Characterization of Activated Carbon Samples

For all activated carbons from this study, nitrogen adsorption isotherms were determined at −196 °C using an automatic equipment ASAP 2020 (Micromeritics, Norcross, GA, USA) after outgassing the samples at 110 °C under secondary vacuum for at least 48 h. Based on the adsorption isotherms, the following information was obtained: the values of classical parameters related to the porous structure of activated carbons, i.e., the specific area *A_BET_* calculated from the BET equation [36]; the volume of micropores, *V_DR_*, calculated from the DR equation [37]; the total pores volume, *V_T_*, calculated from the maximum amount of nitrogen adsorbed at *P*/*P*_0_ = 0.98; and the volume of mesopores, *V_meso_*, calculated as *V_T_* − *V_DR_*; the values of parameters of the porous structure from the LBET method, i.e., *V_hA_*, *Q_A_*/*RT*, *B_C_*, *α*, *β,* and the value of the surface heterogeneity parameter, *h* [38,39,40,41]; the adsorption energy distributions on the first layer, obtained by the LBET method; and the pore size distributions obtained for activated carbons analyzed using the QSDFT [42,43] and 2D-NLDFT-HS methods [44,45,46].

## 3. Results and Discussion

**Figure 1:**
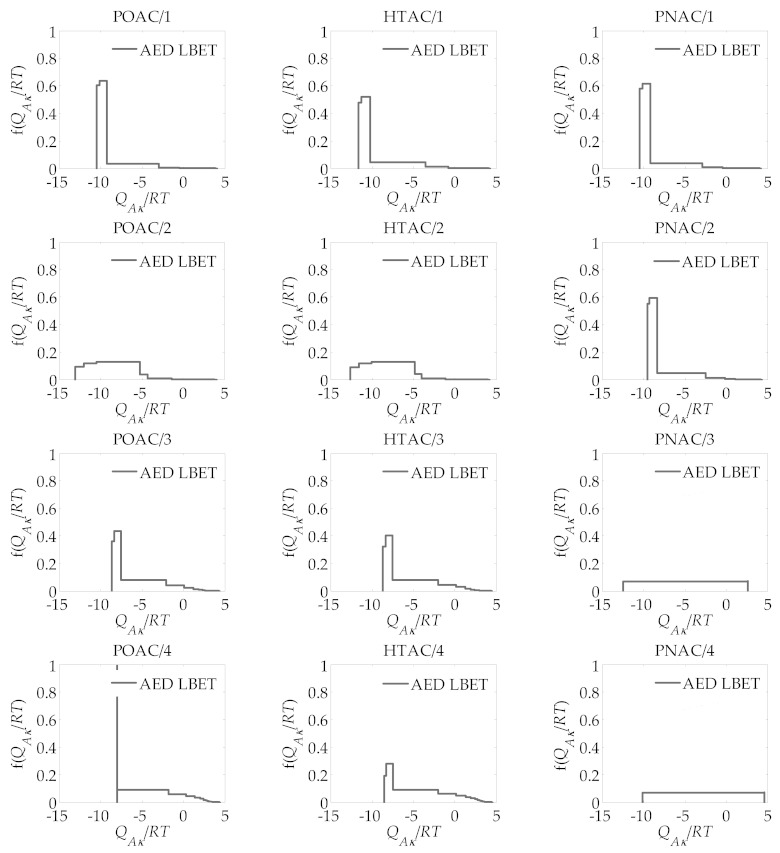
Adsorption energy distributions obtained for all analyzed activated carbons, using the LBET method applied to their nitrogen adsorption isotherms at −196 °C.

**Figure 2:**
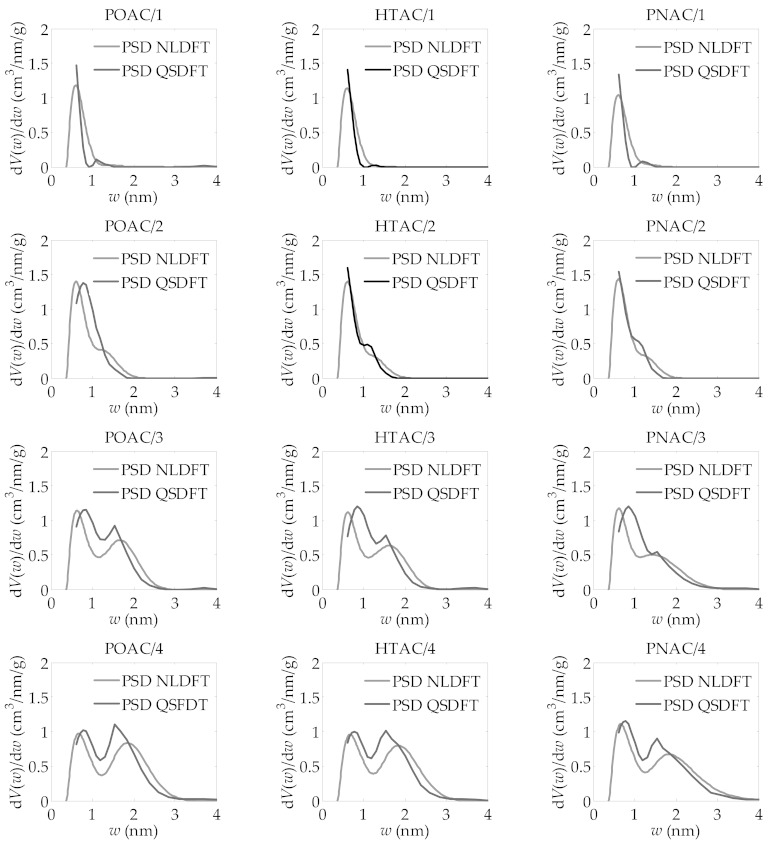
Pore size distributions obtained for all analyzed activated carbons, using the QSDFT and 2D-NLDFT-HS method applied to their nitrogen adsorption isotherms at −196 °C.

The textural parameters obtained from the application of the BET and DR methods are presented in Table 1, whereas those derived from the LBET method are given in Table 2. The corresponding adsorption energy distributions of all activated carbons are shown in Figure 1, and their pore size distributions obtained by application of the QSDFT and 2D-NLDFT-HS methods are presented in Figure 2.

As expected, the results of the analysis from nitrogen adsorption isotherms presented in Table 1 confirm the very high potential of biomass waste for the production of activated carbons using potassium hydroxide. These activated carbons are indeed characterized by very high specific areas and large micropores volume. It is also confirmed that an increase in the mass ratio of activator to char leads to an increase of the specific area *A_BET_* of the activated carbons obtained, their micropores volume *V_DR_*, and their total pores volume *V_T_*. However, with the increase in *R* value, the contribution of the mesopores volume *V_meso_* to the total porosity of all analyzed samples increased. The highest values of *A_BET_*, as well as *V_DR_*, *V_meso_*, and *V_T_* parameters were thus obtained for activated carbons produced with an activator to char mass ratio *R* = 4. In particular, the highest micropores volume was obtained for activated carbon produced from pecan nut shells and designated as PNAC/4 (*V_DR_* = 1.048 cm^3^/g), and the highest specific area was obtained for activated carbon produced from pistachio shells, designated as POAC/4 (*A_BET_* = 3240 m^2^/g).

The results obtained from the BET and DR methods showed that the values of *A_BET_*, *V_T_*, *V_micro_*, and *V_meso_* are very similar for individual activated carbons prepared at the same mass ratios, which may mistakenly suggest that these materials have similar adsorption properties. This is why the conducted research used the LBET method to analyze the adsorption isotherms. It turned out that only such kind of analysis gives a much broader spectrum of information on the porous structures investigated, and thus about the real effect of the mass ratio of activator to char *R* on the development of the porous structure.

Regarding the activated carbons derived from pistachio shells (POAC/*R*), in the case of the sample POAC/1 obtained at *R* = 1, a large value of the parameter *V_hA_*, 0.426 cm^3^/g, was found, and the corresponding LBET model number indicates cluster growth restrictions due to pore geometry limitations and high adsorption energy in layers *n* > 1. This is confirmed by the values of the dimensionless energy parameters *Q_A_*/*RT* = −10.43 and *B_C_ =* 7.98, as well as by the values of the dimensionless geometric parameters *α* = 0.25 and *β* = 1.00, indicating that low and unbranched clusters of adsorbate molecules are formed in the pores. The value of the parameter *h* also indicates that the surface of the activated carbon under study is strongly heterogeneous. The shape of the surface adsorption energy distribution determined for the sample POAC/1 also suggests a relatively narrow spectrum of high-energy adsorption sites and a large fraction of low-energy sites.

The next sample of activated carbon analyzed marked as POAC/2, obtained with the ratio *R* = 2, had a significantly higher volume of the first absorbed layer *(V_hA_* = 0.731 cm^3^/g). The number of the best performing LBET model indicates the restrictions on the growth of adsorbate molecule clusters related to competitive adsorption, and the higher value of the *Q_A_*/*RT* energy parameter (*Q_A_*/*RT* = −13.10) suggests high adsorption interactions in the first adsorbed layer. The values of the geometric parameters *α* and *β* did not change significantly from those of the sample POAC/1 obtained at *R* = 1. However, there was an increase in the value of the parameter *h*, and thus a significant increase in the surface heterogeneity. The shape of the adsorption energy distribution on the first layer also indicates the occurrence of a wide energy spectrum of the adsorption sites on the surface of the adsorbent under study, and thus a significant energy heterogeneity of the surface.

The values of the parameters *V_hA_* and *α* were almost doubled in the sample POAC/3 prepared with *R* = 3 compared to the previously analyzed material. This sample also had a lower value of the parameter *h* compared to the activated carbon POAC/2 obtained at *R* = 2. The shape of the adsorption energy distribution for the first absorbed layer indicates the predominant contribution of primary high-energy adsorption sites.

The sample POAC/4 was characterized by the largest volume of the first adsorbed layer (*V_hA_* = 1.434 cm^3^/g) and the greatest height of the adsorbed clusters (*α* = 0.75), with the lowest degree of surface heterogeneity (*h* = 3). The distribution pattern of adsorption energies for the first adsorbed layer indicates the dominant contribution of a narrow spectrum of sites with high adsorption energy.

For activated carbons obtained from hazelnut shells (HTAC/*R*), for individual activated carbon samples obtained at the same mass ratios *R*, almost identical results were obtained to those of the above-mentioned activated carbons obtained from pistachio shells (POAC/R) therefore, they will not be analyzed or discussed in greater detail. Different results were obtained for activated carbons from pecan nut shells (PNAC/*R*). While for the ratio *R* = 1, similar results to those of POAC/*R* and HTAC/*R* activated carbons were obtained, at *R* = 2, a lower degree of surface heterogeneity was observed, and the largest differences were observed for *R* = 3 and *R* = 4. Significant differences can be observed in the case of PNAC/3, namely that the surface of this material was found to have the lowest degree of heterogeneity (*h* = 1), and its surface is uniformly heterogeneous, as also indicated by the shape of the adsorption energy distribution. The values of the geometric parameters *α* and *β* indicate that only single nitrogen molecules are adsorbed in the pores of this material. It should be stressed that this is a desirable feature in many technological processes, as such a material exhibits molecular sieve properties, adsorbing only molecules of a certain size. In turn, the activated carbon sample PNAC/4 was characterized by the largest volume of the first adsorbed layer among all the activated carbons investigated here (*V_hA_* = 2.131 cm^3^/g), and small non-branching clusters of adsorbate particles were formed in its pores. The PNAC/4 sample was also characterized by a very low degree of heterogeneity (*h* = 1), a feature desired in most advanced adsorption processes.

The values of *V_DR_* and *V_T_* showed that activated carbons obtained at mass ratios of activator to product of the carbonization process *R* = 3 and 4 are characterized by a significant share of mesopores in the total porosity. However, based on the information obtained by BET, DR, and LBET methods, it is not possible to deduce which pore size ranges are present in the analyzed activated carbons. Therefore, the pore size distributions were determined for all activated carbons using the QSDFT and 2D-NLDFT-HS methods, i.e., taking into account the heterogeneity of the adsorbent surface.

Based on the results of the QSDFT and 2D-NLDFT-HS calculations presented in Figure 2, very similar pore size distributions can be seen for the activated carbons obtained from different materials under identical preparation conditions. These distributions are essentially bimodal, i.e., there are two peaks on the distributions corresponding to the dominant micropores’ contributions in the range of about 0.5 nm to 1 nm and in the second from about 1 to about 2 nm. Moreover, it can be observed that the fraction of micropores in the range of 1 to 2 nm increases with the ratio of activator to char.

The analysis performed by the QSDFT and 2D-NLDFT-HS methods, in particular the detection of the bimodal pore structure, provided new information impossible to obtain by the BET, DR, or LBET methods. Especially when the activator to char mass ratio *R* increases, the porous structure of the samples develops, mainly in the pore range of about 1 to 3 nm, with small changes in the range of about 0.5 to 1 nm. Therefore, as *R* increases, larger micropores and mesopores correspond to an increasing fraction of the porosity and, at *R* = 4, this proportion is dominant.

The 2D-NLDFT-HS method, however, as can be seen in Figure 2, does not cover the full range of narrow micropores in the same way as the classical NLDFT method, therefore the QSDFT method is more useful, despite the adoption of the model of one-dimensional surface heterogeneity.

## 4. Conclusions

The results of the research presented in this article have highlighted the significant potential of the shells of pistachio, hazelnut, and particularly pecan nuts for the production of activated carbons by preliminary carbonization followed by activation with potassium hydroxide. The most developed and homogeneous porous structures were obtained from pecan nut shells. As this paper has shown, food industry waste is a cheap source of raw material for the production of high-quality activated carbons, which can be used in many industrial applications and in everyday life.

Based on the analysis of nitrogen adsorption isotherms, the differences in the adsorption properties of these materials, significant from the point of view of their practical application, were demonstrated using the LBET method. The latter made it possible to determine not only the adsorption energy on the adsorbent surface, but also on the subsequent adsorbed layers. It was thus possible to calculate with which energy the adsorbate molecules are adsorbed and for which type of adsorption there are preferential energy conditions, i.e., monolayer or multilayer adsorption. In addition, the shape and size of the clusters of adsorbate molecules formed in the pores were inferred from the shape and size of the micropores, as well as small mesopores. Significant information about the texture of the adsorbents was also obtained from the surface heterogeneity index values.

The conducted research also indicates that the results obtained using the BET and DR methods correlate very well with those obtained by the LBET, QSDFT, and 2D-NLDFT-HS methods. Bearing in mind the important limitations of these methods, they can be considered useful in the analysis of the influence of preparation conditions on the formation of the structure of porous carbon adsorbents. However, in order to obtain reliable information on the porous structures and the influence of the preparation conditions, it is recommended to use the LBET and QSDFT or 2D-NLDFT-HS methods simultaneously and, in addition, BET and DR. Only such an approach provides a full spectrum of reliable information on the materials analyzed and allows for the optimum selection of preparation conditions to obtain activated carbons with strictly defined adsorption properties from a specific raw material.

## Figures and Tables

**Table 1:** Basic parameters of the porous structure of activated carbons calculated from nitrogen isotherms obtained at the temperature of −196 °C.

*R*	*A_BET_* m^2^/g	*V_DR_* cm^3^/g	*V_T_* cm^3^/g	*V_meso_* cm^3^/g
POAC/*R*				
1	1109	0.478	0.508	0.030
2	2004	0.801	0.893	0.092
3	2911	0.964	1.337	0.373
4	3240	0.990	1.568	0.578
HTAC/*R*				
1	1063	0.461	0.476	0.015
2	1804	0.747	0.810	0.063
3	2687	0.903	1.233	0.330
4	3163	0.984	1.529	0.545
PNAC/*R*				
1	1017	0.438	0.451	0.013
2	1845	0.762	0.827	0.065
3	2493	0.884	1.184	0.300
4	3183	1.048	1.630	0.582

**Table 2:** Parameters from the analysis of the microporous structure of activated carbons, based on the LBET method applied to nitrogen adsorption isotherms at −196 °C.

*R*	LBET No.	*V_hA_* cm^3^/g	*Q_A_*/*RT*	*B_C_*	*h*	*α*	*β*
POAC/*R*							
1	22	0.426	−10.43	7.98	5	0.25	1.00
2	15	0.731	−13.10	7.51	9	0.27	1.00
3	22	1.329	−8.62	6.92	5	0.61	1.00
4	19	1.434	−8.02	7.23	3	0.75	1.00
HTAC/*R*							
1	22	0.443	−11.62	7.96	5	0.35	1.00
2	15	0.811	−12.62	7.23	9	0.30	1.00
3	22	1.225	−8.65	6.26	5	0.64	1.00
4	22	1.489	−8.56	5.66	5	0.79	1.00
PNAC/*R*							
1	22	0.407	−10.40	7.66	5	0.28	1.00
2	22	0.743	−9.54	7.66	5	0.35	1.00
3	2	1.350	−12.41	6.04	1	0.00	1.00
4	2	2.131	−10.12	7.66	1	0.33	1.00

## Data Availability

The data presented in this work can be made available on request.
